# The “Hot Cross Bun Sign” in Spinocerebellar Ataxia Types 2 and 7–Case Reports and Review of Literature

**DOI:** 10.1002/mdc3.13550

**Published:** 2022-10-13

**Authors:** Ansuya Kasavelu Naidoo, Cait‐Lynn Deanne Wells, Yashvir Rugbeer, Neil Naidoo

**Affiliations:** ^1^ Greys Academic Hospital Pietermaritzburg South Africa; ^2^ School of Clinical Medicine, Division Neurology University of KwaZulu Natal Durban South Africa

**Keywords:** hot cross bun sign, spinocerebellar ataxia type 2 and 7, multiple system atrophy (MSA)

## Abstract

**Background:**

The “hot cross bun” sign is a cruciform hyperintensity is seen on T2 weighted imaging within the pons. The sign is considered to be pathognomic for Multiple system atrophy type C. The clinical and radiological features of Multiple system atrophy type C overlap with the autosomal dominant inherited ataxias. We present a case series of 3 African patients with genetically proven Spinocerebellar Ataxia presenting with the Hot cross bun sign and a scoping review of similar studies.

**Cases:**

We described the phenotypic and radiological presentation of genetically confirmed SCA‐2 in two, and SCA‐7 in one patient, with the “hot cross bun” sign.

**Literature Review:**

We performed a scoping review on the Hot Cross Bun Sign.

A total of 66 articles were retrieved. We describe the diverse aetiologies of the sign and associated phenotypic and radiological features. We review the Spinocerebellar Ataxias described with a Hot cross bun sign and make comparisons to Multiple System Atrophy Type C [Ref. 1,2].

**Conclusions:**

To our knowledge this is the first description of an African cohort presenting with the Hot Cross Bun Sign. We expand the differential diagnosis of the Hot Cross Bun Sign.

## Introduction

The “hot cross bun” sign (HCBS) is a radiological description on axial T2W brain magnetic resonance imaging (MRI), of a cruciform hyperintensity within the pons. It is pathognomic for Multiple system atrophy type C (MSA‐C) and is also described amongst the autosomal dominant heredo‐degenerative ataxias.

The Spinocerebellar ataxias (SCAs) are a heterogeneous group of ataxias characterized by cerebellar and spinal cord degeneration. The annual incidence is estimated at 2.02/10000000 per year in South Africa.^1^, ^2^ Multiple System Atrophy (MSA) is a neurodegenerative disorder, characterized by glial alpha‐synuclein inclusions. It occurs sporadically in males and females older than 60 years. Patients present with a cerebellar, parkinsonian or autonomic subtype characterized by olivopontocerebellar degeneration. Clinical hallmarks include symmetrical parkinsonism, absent tremor, autonomic failure, ataxia, pyramidal signs and vertical gaze ophthalmoparesis. The HCBS is unique to MSA‐C.

There is significant clinical and radiological overlap between MSA‐C and the SCAs. The reliability of the HCBS as a diagnostic marker in differentiating the two conditions is obscure. There are isolated HCBS descriptions in several unrelated conditions, suggesting it should no longer be considered diagnostic of MSA‐C.

We present a series of African patients with genetically confirmed SCAs and a HCBS, which has not previously been described in this population. We review the literature on the topic and discuss the differential diagnosis.

## Case 1

A 28 year‐old Black female with no chronic medical illnesses, drug, alcohol or toxin exposure, presented with an 11 year history of progressive in‐coordination. The history was suggestive of an inherited ataxia with the patient's father having demised of a similar condition. Clinical examination revealed spasticity in both lower limbs, saccadic eye movements on smooth pursuit, profound dysarthria, and a pan‐cerebellar syndrome with gait ataxia. The rest of the examination was normal.

**FIG. 1:**
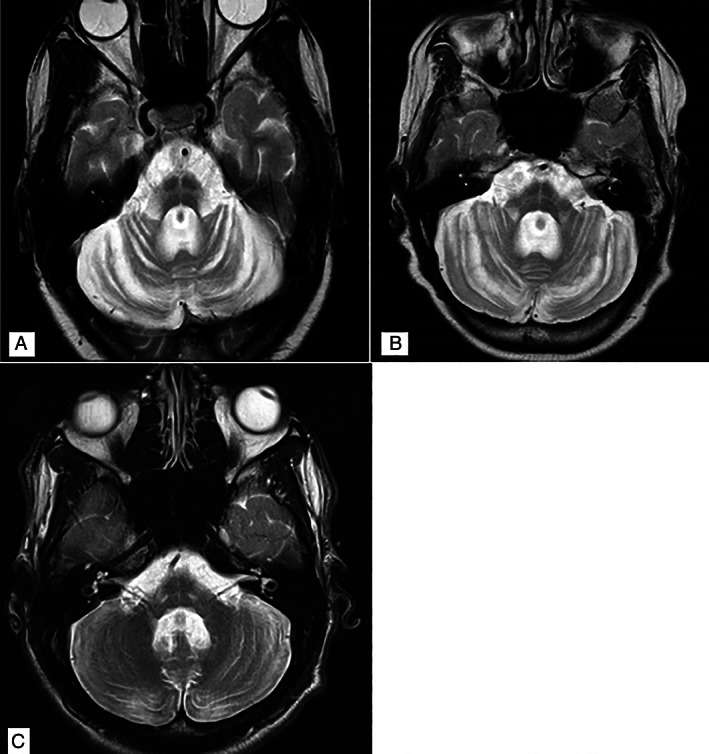
MRI T2W Axial MRI at mid‐pontine level of Case 1 (A), Case 2 (B) and Case 3 (C). A cruciform hyperintensity with atrophy of the brainstem, cerebellum and bilateral MCP hyperintensities are noted in (A, B) and asymmetrically in (C).

Laboratory investigations including HIV Elisa, infective, paraneoplastic, autoimmune and metabolic screens and cerebrospinal fluid (CSF) examination were normal. Genetic testing revealed an expansion mutation at the *ATXN2* (SCA2) gene locus. One normal and one fully expanded allele were observed (21/47). MRI brain showed a HCBS with cerebellar and brainstem atrophy and a bright middle cerebellar peduncle (MCP) sign (Fig. 1) and Table 1.

**TABLE 1:** Causes of the HCBS described in the scoping review

Causes of HCBS Described in the Scoping Review			
Reported Aetiologies	Number of Retrieved Reports	Reported SCAS with HCBS	Number of Retrieved Reports
Parkinsonian Syndromes		SCA Type	
Multiple System Atrophy – C	40^20^, ^40^, ^42^, ^43^, ^44^, ^46^, ^48^, ^49^, ^50^, ^51^, ^53^, ^54^, ^55^, ^61^, ^62^, ^63^, ^64^, ^65^, ^66^, ^67^, ^68^, ^69^, ^70^, ^71^, ^72^, ^73^, ^74^, ^75^, ^76^, ^77^, ^78^, ^79^, ^80^, ^81^, ^82^	SCA 1	6^5^, ^7^, ^83^, ^84^, ^85^, ^86^
Multiple System Atrophy–P Multiple System Atrophy‐ A	2^50^, ^51^	SCA 2	11^6^, ^7^, ^8^, ^18^, ^44^, ^52^, ^84^, ^85^, ^86^, ^87^
Probable Dementia with Lewy Body	1^15^	SCA 3	9^3^, ^5^, ^6^, ^12^, ^44^, ^84^, ^86^, ^88^, ^89^
Corticobasal Degeneration (olivopontocerebellar type)	1^14^	SCA 6, 7, 8	17^4^, ^6^, ^44^, ^47^, ^84^, ^86^, ^90^, ^91^
Parkinsonism Related Vasculitis	1^16^	SCA 10	1^84^
Infection		SCA 17	1^10^
Rabies Encepaltiis	1^2^	SCA 23	1^92^
HIV related Progressive Multifocal Leukoencephalopathy	4^22^, ^23^, ^25^, ^58^	SCA 31	1^47^
Natalizumab associated Progressive Multifocal Leukoencephalopathy	1^29^	SCA 34	4^13^, ^86^, ^93^, ^94^
HIV related JCV granule cell neuronopathy	1^60^	SCA 42	1^95^
HIV	2^27^, ^28^		
Brainstem encephalitis	2^19^, ^20^		
Neoplastic			
Paraneoplastic Rhombencephalitis	2^27^, ^30^		
Leptomeningeal malignant involvement in Breast Cancer and Melanoma	2^31^, ^32^		
Lung Cancer Undefined Cause	1^20^		
Kelch‐like protein 11‐associated paraneoplastic neurological syndrome associated seminoma	2^29^, ^33^		
Vascular			
Bilateral middle cerebral peduncle infarction	1^17^		
cerebellar hemorrhage (n=1). 16‐18 (n=1). 16‐18(n=1). 16‐18	1^18^		
Neurodegenerative			
Variant Creutzfeldt Jakob Disease	1^26^		
Fragile X tremor ataxia syndrome	1^39^		
Cerebrotendinous xanthomatosis	1^37^		
Non infective Inflammatory			
Neurosarcoidosis	1^36^		
Autoimmune cerebellar ataxia—Homer‐3 antibodies.	1^34^		
ADEM, NMO. MS,	1^20^		
Toxin			
Toxic Encephalopathy with Phenytoin Sodium	1^20^		
Other			
Oculodentodigital dysplasia	1^96^		
Myaesthenia Gravis	1^35^		

Abbreviations: ADEM, Acute Disseminated Encephalomyelitis; HCBS, Hot Cross Bun Sign; HIV, Human Immunodecficiency Syndrome; JCV, John Cunningham Virus; KELCH, Kelch like protein‐11; MS, Multiple Sclerosis; NMO, Neuromyelitis Optica; SCA, Spinocerebellar Ataxia.

## Case 2

A 44 year‐old Black female, known with HIV infection and on anti‐retroviral therapy, presented with a 4 year history of head tremor and in‐coordination. Medication, alcohol, toxin and family history were unremarkable. Clinically she had a normal motor and sensory examination, a pan‐cerebellar syndrome and an ataxic gait. Laboratory testing for infective, autoimmune and metabolic disorders and CSF examination including *Varicella Zoster Virus, Herpes Simplex Virus, Cytomegalovirus* and *John Cunningham Virus (JCV)* PCR were normal. Her CD4 count was 51 cells/mm3. Genetic testing revealed an expansion mutation at the *ATXN2* (SCA2) gene locus. One normal and one fully expanded mutation were observed (21/40). MRI showed a HCBS, atrophy and MCP hyperintensities (Fig. 1).

## Case 3

A 21 year‐old Black male with no co‐morbidities, presented with 4 year progressive history of unsteady gait, dysarthria and poor vision. Family history was positive; his father had developed progressive gait imbalance at 45 years of age. Clinical examination revealed dysarthria, total ophthalmoparesis, reduced visual acuity bilaterally with a retinal dystrophy on slit lamp examination. He was quadrispastic, hyperreflexic, with upgoing plantar responses and a length dependent sensory neuropathy. He demonstrated a pan‐cerebellar syndrome with truncal and gait ataxia. Laboratory investigations for paraneoplastic and autoimmune screens, alpha‐foeto protein levels and CSF examination were normal. MRI Brain demonstrated a HCBS with atrophy, and a Bright MCP sign on the right (Fig. 1). Genetic testing confirmed a CAG repeat expansion at the ATXN7 (SCA 7) gene locus. One normal and one fully expanded allele were observed (11/57).

## Literature Review

**FIG. 2:**
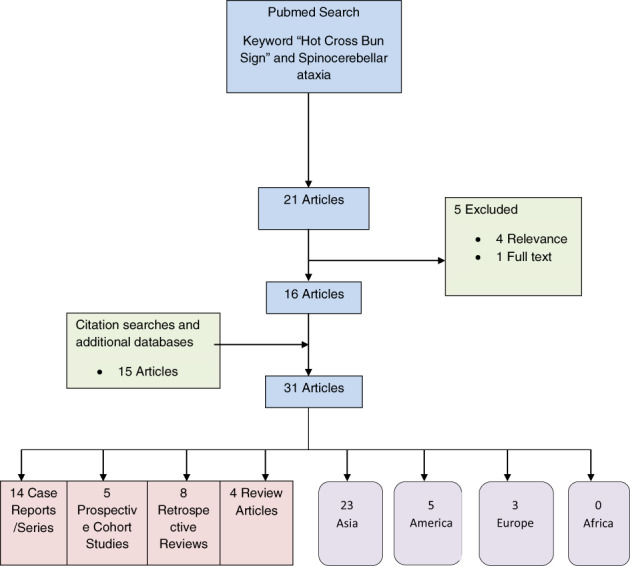
Flow diagram of the scoping review on the hot cross bun sign and spinocerebellar ataxia.

**FIG. 3:**
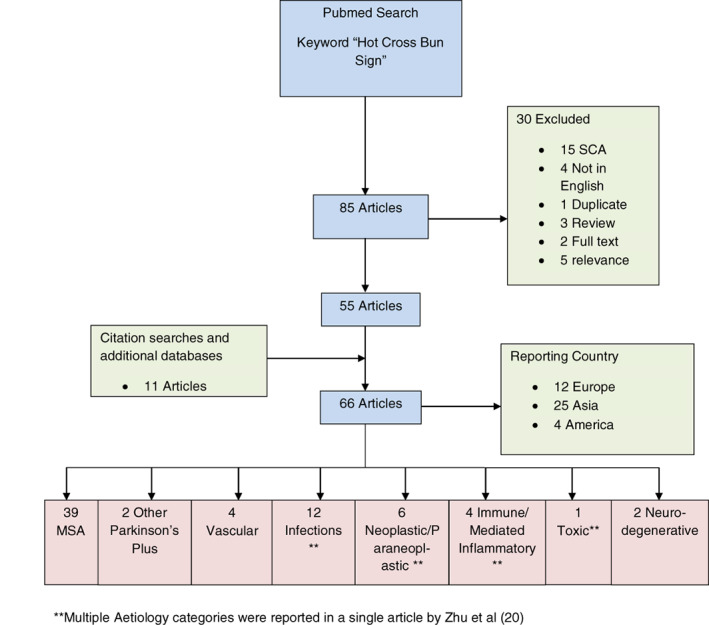
Flow diagram of the scoping review on the hot cross bun sign.

The cases presented raise questions of “What is the diagnostic utility of the HCB sign?” and “Has the HCBS been described in an African cohort of patients?”. We conducted a scoping review of the literature to evaluate for articles reporting on SCA cases with a HCBS and the HCBS in other conditions. A broad literature search was conducted for relevant reports indexed in Pubmed/Medline. The keywords "Hot Cross Bun Sign" and "Spinocerebellar Ataxia" were used. Studies were excluded if they were not (i) relevant, (ii) available in English or (iii) as full text. Title and abstract screening and review of eligible articles were performed. The extracted data are summarized in Figures 2 and 3 and Tables S1 and S2.

The HCBS is well described amongst the SCAs (Table 1). There are descriptions in SCA ‐1, −2, −3, −7, −8 and − 34.^3^, ^4^, ^5^ Asian studies report a prevalence of 8.7% and frequency of 12.8% of the HCBS in patients with SCA, independent of the subtype. No data for the African continent was available. Other SCAs that demonstrated the HCBS include SCA‐3, −6 and infrequently SCA‐1, −7 and − 8. A significant association with SCA‐2 is described with a specificity of 97.7%.^6^, ^7^ Lee et al found a prevalence of 25.7% in SCA‐2, 1.3% in SCA‐3 and isolated cases of SCA‐7 and ‐8.^6^ There are several descriptions of SCA‐2 and the HCBS in our review (n = 11).^8^, ^9^ We retrieved cases describing SCA‐7 (n = 6), SCA‐3 (n = 9), SCA‐17 (n = 1) and SCA‐6 (n = 5), SCA‐8 (n = 6). SCA‐7 was not frequently associated with the HCBS. Our review included SCA ‐23, −31, −34 and − 42 (n = 7).^10^ SCA −34 cases (*ELOVL4* mutations) reported the HCBS in 66.7% of patients.^11^, ^12^, ^13^

The age range of HCBS in other conditions was 3–85 years of age, with a median age of 57.2 years. There were 30 males and 25 females. The sign is reported in MSA‐C, (n = 39) including one presentation with MSA‐P (Table 1). There were 2 reports in other Parkinson's Plus conditions included an autopsy confirmed case of Corticobasal Degeneration (CBD), a probable case of Dementia with Lewy Bodies and a case of vasculitis induced parkinsonism.^14^, ^15^, ^16^ The case of CBD in an olivopontocerebellar phenotype is the first description of the HCBS in CBD. A diagnosis of CBD was based on cortical features, cognitive decline and extrapyramidal signs. The appearance of the HCBS was delayed 5 years after symptoms onset.^14^

Vascular aetiologies included bilateral pontine infarction (n = 1), vasculitis (n = 1) and cerebellar hemorrhage (n=1).^16^, ^17^, ^18^

Infective aetiologies included viral encephalitis in a child, infective brainstem encephalitis and acute stage rabies encephalitis.^19^, ^20^, ^21^ There were 7 cases of PML associated HCBS, 1 presenting as a JCV neuronopathy.^22^, ^23^ All cases were associated with HIV infection except one following Natalizumab use in a Multiple Sclerosis (MS) patient.^24^ These authors described a rostrally appearing linear hyperintensity. They coined the term “across the pons sign” and proposed this is a distinguishing feature of PML related HCB. HIV infection itself with advanced disease (CD4 counts below 200 cells/mm^3^) (n = 4) was described and Variant Creutzfeld Jakob disease (v‐CJD) was reported in an isolated case with HCBS.^25^, ^26^, ^27^, ^28^

Neoplastic associations included paraneoplastic neurological syndromes (n = 2), leptomeningeal carcinomatosis in breast cancer and melanoma (n = 2) and lung cancer (n = 1).^20^, ^29^, ^30^, ^31^, ^32^ Paraneoplastic antibodies with anti‐Amphiphysin, (presenting as a rhombencephalitis in breast cancer) and Kelch like protein‐11 (in two patients with a background of seminoma) are noted.^29^, ^30^, ^33^ Autoimmune aetiologies include 2 cases of ataxia secondary to Homer‐3 antibodies and a delayed appearance of the HCBS by 24 months.^34^ Patients had a resemblance to a MSA‐C phenotype and were treated with immunotherapy, with a partial response.

Other inflammatory non‐infective disorders demonstrating the HCBS were neurosarcoidosis (n = 1) and Myaesthenia Gravis (n = 1).^35^, ^36^ Zhu et al in a systematic review described the HCBS in inflammatory demyelinating disorders: Neuromyelitis Optica Spectrum Disorders, MS and Acute Disseminating Encephalomyelitis.^20^, ^36^

A single case of toxic encephalopathy with Phenytoin, on a background of cerebellar atrophy is described.^20^ The HCBS has been described in neurodegenerative conditions of cerebrotendinous xanthomatosis, in a consanguineous family and in the Fragile X Tremor Syndrome (FTAXS).^37^, ^38^, ^39^

There were no studies from Africa or reports of patients of African descent with SCA and HCBS in the conditions retrieved.

Ataxia was the commonest clinical sign amongst cases regardless of underlying aetiology. Parkinsonism and dysautonomia occurred consistently in patients with MSA‐C and were less likely to occur in non‐Parkinsonian aetiologies. All patients presented with a cruciate hyperintensity of the pons best seen on T2W or T2 FLAIR sequences which were graded radiologically into four grades.^20^ Other imaging findings include the inverse trident sign in 2 cases‐neurosarcoidosis and an HIV infected patient with PML, as well as the “across the pons sign” in Natalizumab induced PML.^22^, ^23^

The HCBS in MSA‐C has a sensitivity of 76.9%–87.5% with a specificity of 94.6%–100% in differentiating MSA‐C from the SCAs.^40^, ^41^, ^42^ Associated radiological features in MSA‐C include extension into the upper medulla and atrophy of the cerebellum, pons and MCP. Atrophy was common in degenerative and hereditary disorders, especially in MSA‐C (n = 28). Atrophy was also present in post vascular events (n = 2), HIV infected patients regardless of PML infection (n = 4) and in autoimmune antibodies disease with Homer‐3 and Kelch like protein 11 (n = 2), leptomeningeal metastases (n = 1). A notable lack of atrophy was seen in anti‐Amphiphysin paraneoplastic syndrome and post viral encephalitis.

The “Bright MCP sign” was seen consistently in MSA‐C (n = 12), HIV infection and PML (n = 4), paraneoplastic rhombencephalitis (n = 2) and one case of Parkinsonism secondary to vasculitis n = 1. It was not described in any of the other aetiologies listed. In our case series 2 patients presented with bilateral MCP hyperintensities and 1 with more prominent Bright MCP on one side.

Imaging findings in MSA‐C were pontocerebellar and MCP atrophy, ex‐vacuo dilatation of the fourth ventricle, MCP hyperintensity and the HCBS.^43^, ^44^, ^45^ These features are useful in differentiation from Parkinson's disease.^46^ The HCBS is highly specific early in the MSA‐C course.^3^ Comparative studies with the SCAs show that the HCBS occur in MSA‐C (within 3 years of onset), correlating with prominent proinflammatory markers, intrathecal inflammation and radiological progression on longitudinal follow‐up.^47^

The “Putaminal rim sign” which is a hallmark of MSA‐P is also described in MSA‐C and appears with disease progression.^42^, ^48^, ^49^ Conversely the HCBS may appear before the hallmark putaminal sign in MSA‐P and occurred in MSA‐A (n = 1).^50^, ^51^

Our case series generates the question as to why is there is a variable presentation of the HCBS amongst the SCAs? The HCBS occurs independent of repeat expansion length; with age related atrophy contributing to its prominence and the HCBS grade differing depending on SCA subtype.^5^, ^6^ Significant atrophy occurs in SCA‐2 and ‐7 and may contribute to the increased prevalence in SCA‐2. SCA‐6 demonstrates minimal atrophy and does not feature the HCBS or a vertical midline linear hyperintensity. The Grade 1 HCBS is seen in SCA‐1.^5^

Cerebellar atrophy is the commonest imaging finding amongst the SCAs and was present in all SCA cases reviewed^4^ SCA‐2 demonstrates olivopontocerebellar atrophy, pallor of the substantia nigra and frontal lobe atrophy. Radiological changes in SCA‐2, the MCP hyperintensity and HCBS, appeared after 5 years disease duration.^44^ Radiological descriptions of SCA‐34 included atrophy, vertical linear pontine hyperintensity and a hyperintense MCP in 33% of patients.^11^, ^12^, ^13^

The commonest clinical presentation was ataxia in all patients, with autonomic dysfunction (n = 13) and urinary symptoms (n = 11) noted.^52^ The phenotype of SCA‐34 mimicked MSA‐C. There were significant phenotypic, radiological and pathological similarities between SCA‐2 and MSA‐C.

## Discussion

We present three cases of genetically confirmed SCAs in a South African setting that demonstrate the HCBS. MSA‐C and SCA‐2 may be indistinguishable clinically and radiologically with the exception of disease duration.

The HCBS reflects atrophy and selective loss of myelinated transverse pontocerebellar fibers in neurodegenerative conditions and Wallerian degeneration or gliosis following vascular events.^17^, ^18^, ^53^ This accounts for the delayed appearance of the sign.^17^, ^20^, ^54^ The pontine tegmentum and corticospinal tracts are spared.^41^ The HCBS reflects the underlying pathological process in MSA‐C and SCA‐2. Longitudinal imaging studies suggests that an increasing grade of HCB is an indicator of increasing disease severity in MSA‐C.^20^, ^55^

The pathogenic mechanism of the HCBS may differ based on the underlying disease. In v‐CJD neuronal loss, astrocytosis, plaques with spongiform changes in pontine nuclei and degeneration of pontocerebellar tracts were described. In cases of PML and HIV infection involvement of the granule cell layer is implicated.^56^ The lack of atrophy in post encephalitis cases with radiological recovery following immunomodulatory treatment suggests alternative neuro‐inflammatory mechanisms for its appearance.^20^

The HCBS has a predilection for SCA‐2 and occurred at a higher frequency in our review.^21^ In SCA‐2 and MSA‐C atrophy of the pons is prominent, sparing the pontine tegmentum and the corticospinal tracts with bilateral involvement of the MCPs.^9^, ^41^, ^44^, ^57^ The HCBS and bright MCP sign occur at a frequency of 13.6% in SCA‐2 and is not described in the other SCAs.^44^ We describe it occurring in our cases of SCA‐2 and SCA‐7. The MCP is a major input tract into the cerebellum and the reason for its selective involvement is unclear.

Clinical features of SCA‐2 may be heterogeneous.^9^ Our SCA‐2 patients did not demonstrate the characteristic saccadic, ophthalmoplegic or cognitive abnormalities described.^1^, ^2^

A neuropathological analysis of SCA noted Lewy bodies and Lewy neurites consistent with an alpha synucleinopathy in a single patient.^15^ MSA‐C is an alpha‐synucleinopathy. The possibility of neuropathological overlap may be an explanation for the clinical and radiological overlap of both conditions.

Our scoping review has widened the spectrum of SCAs and aetiologies reported with the HCBS and lend support to a broad differential diagnosis beyond MSA‐C.

We found no South African reports of the HCBS in HIV infected patients despite the high burden of HIV infection. The HCBS is reported in HIV associated PML and HIV associated ataxia with asymmetrical hyperintensities described in PML.^27^, ^58^, ^59^ One patient in our series was HIV and SCA‐2 positive. A granule cell neuronopathy was excluded and no asymmetry of hyperintensities was noted. Longitudinal analysis of HIV infected patients with JCV demonstrated the HCB sign had a delayed appearance even in burnt out JCV infection, indicating HIV progression or retrograde neuronal loss with gliosis.^27^, ^58^, ^60^ The appearance of the HCBS in an HIV infected cohort will be worth investigating.

Our study is limited by being a case series of patients who presented to a tertiary neurology facility for investigation and noted to have a HCBS. A retrospective review of all SCA ‐, MSA ‐ or HIV infected patients who presented to the center was not analyzed. The strength of this study is that we conducted a scoping review on multiple databases and expanded the differential diagnosis of the HCBS. We provide the first reported case series of the HCBS in an African cohort with a similar phenotype and radiological presentation as reported in the literature.

### Conclusion

The widened differential of the HCBS suggests this is a radiological indistinct entity with an underlying pathological process of Wallerian degeneration of the pontocerebellar fibers rather than a disease specific pathological process. It should no longer be considered pathognomic of MSA‐C.

We describe the HCBS in a cohort of Black South African patients and demonstrate phenotypic and radiological concordance with descriptions in other parts of the globe.

## Author Roles

(1) Research project: A. Conception, B. Organization, C. Execution; D. Design; (2) Manuscript Preparation: A. Writing of the first draft, B. Review and Critique.

A.K.N.: 1A, 1B, 1C, 1D, 2A, 2B.

C.D.W.: 1A, 1B, 1C, 1D, 2A.

Y.R.: 1B, 1C.

N.N.: 1C, 2B.

## Disclosures

**Ethical Compliance Statement:** The study was approved by the Biomedical Research and Ethics Committee (University of KwaZulu Natal) BREC/00002606/2021. Informed patient consent was not necessary for this work. We confirm that we have read the Journal's position on issues involved in ethical publication and affirm that this work is consistent with those guidelines.

**Funding Sources and Conflicts of Interest:** No specific funding was received for this work. The authors declare that there are no conflicts of interest relevant to this work.

**Financial Disclosures for the Previous 12 Months:** The authors declare that there are no additional disclosures to report.

## Supporting information


**Table S1** Scoping review of the Hot Cross Bun Sign in additional conditions

**Table S2** Scoping review of the Hot Cross Bun Sign in the Spinocerebellar Ataxias
