# Supplementary material for: The “Hot Cross Bun Sign” in Spinocerebellar Ataxia Types 2 and 7–Case Reports and Review of Literature
Source: Mov Disord Clin Pract. 2022 Oct 13;9(8):1105–13. doi: 10.1002/mdc3.13550 (PMC9631856; doi:10.1002/mdc3.13550)
Supplement: Supplementary file 1 — Table S1 Scoping review of the Hot Cross Bun Sign in additional conditions [file MDC3-9-1105-s002.doc]

| **Supplementary Table 1. Scoping review of the Hot Cross Bun Sign in additional conditions** | | | | | | | | | | |
| --- | --- | --- | --- | --- | --- | --- | --- | --- | --- | --- |
| **Author** | **Article** | **Place** | **Article Type** | **Size of Cohort** | **Age of Cohort (years)** | **Sex** | **Clinical Phenotype** | **HCB Imaging findings on MRI** | **Additional Imaging Findings** | **Aetiology** |
| 1. Soares-Fernandes (2009) | “Hot Cross Bun” Sign in Variant Creutzfeldt-Jakob  Disease | Portugal | Case Report | N=1 | 16 | F | Persistent Vegetative State | Pontine cruciform hyperintensity  was seen on fluid-attenuated inversion recovery images | Global brain atrophy and widespread restricted  cortical diffusion | Variant CJD |
| 1. Muqit (2001) | “Hot cross bun” sign in a patient with  Parkinsonism secondary to presumed  vasculitis | UK | Case Report | N=1 | 31 | F | Ataxia  Parkinsonism  No dysautonomia | T2W signal  hyperintensity within the pons (hot cross bun  Sign) and high signal change in the middle  cerebellar peduncles | Atrophy of the  medulla, pons, cerebellum, and middle cerebellar  peduncles | Vasculitis |
| 1. Carré et al (2020) | Brain MRI of multiple system atrophy of cerebellar type: a prospective study with implications for diagnosis criteria | France | Prospective study | MSA-C N=26 | Mean age 67.26 | 15 M  11 F | Ataxia  Parkinsonism  Autonomic signs | MCP hyperintensity and HCB T2-proton density weighted | Atrophy of the  midbrain, pons, cerebellum, and middle cerebellar  peduncles Hyperintensity of MCP Hyperintense lateral rim of putamen Hypointense putamen | MSA |
| 1. Kim (2019) | Differential value of brain magnetic  resonance imaging in multiple  system atrophy cerebellar  phenotype and spinocerebellar  ataxias | Seoul Korea | Retrospective review | MSA-C N=186 | Mean age 57.4 | 109 M  77 F | Ataxia  Parkinsonism  Autonomic signs | Hot cross bun signs and middle cerebellar peduncle  hyperintensities were exclusively prevalent in MSA-C compared to SCAs at <3 years | Sensitivity, specificity, and  Positive predictive value (PPV) for HCB sign and MCP hyperintensity for MSA vs SCA was 100% at < 3years disease duration | MSA |
| 1. Schlapakow (2020) | Multiple system atrophy mimicry in MRI: Watch out for paraneoplastic  rhombencephalitis | Germany | Case Report | N=1 | 51 | F | Ataxia  No dysautonomia. | Pontine HCB sign with extension  of the T2-hyperintensities to the MCPs | Pontine lesion showed mild mass effect extending into the midbrain | Amphiphysin antibody-associated paraneo- plastic rhombencephalitis |
| 1. Shrivastava et al (2007) | The Hot Cross Bun Sign | UK | CME Article describing HCB Sign | N/A | N/A | N/A | N/A | Cruciform hyperintenisty seen on transverse T2W imaging of the pons | Atrophy and  Increased signal intensity within the  pons, cerebellum, and MCPs | MSA-C |
| 1. Portet (2019) | Hot Cross Bun Sign | Germany | Case Report | N=1 | 76 | M | Ataxia  No dysautonomia or parkinsonism | Cruciform  hyperintensity in the pons on T2W and FLAIR  Sequences | Pontine and MCP atrophy were also noted, as well as high  signal in the MCPs | MSA-C |
| 1. Cicilet (2017) | Hot cross bun and bright middle cerebellar peduncle signs in cerebellar type multiple system atrophy | India | Case Report | N=1 | 52 | F | Ataxia Orthostatic hypotension. No parkinsonism | Cruciform hyperintense signal in the pons on T2 FLAIR imaging | T2 FLAIR hyperintensity of bilateral MCPs. Cerebellar atrophy | MSA-C |
| 1. Vijayan (2008) | MR imaging in multiple system atrophy: Its role in “splitting” parkinsonism | India | Case Report | N=1 | 59 | F | Ataxia Dysautonomia Parkinsonism. | HCB sign in the pons on T2/ PD/FLAIR imaging | Atrophy of putamen, pons and cerebellum Hyperintensity of bilateral MCPs | MSA-C |
| 1. Schultz (1994) | Multiple system atrophy: natural history, MRI morphology, and dopamine receptor imaging with 123IBZM-SPECT | Germany | Prospective study | N=16 | 40 – 66  Mean 60.1 | 12 M  4 F | Ataxia Dysautonomia Parkinsonism  Pyramidal signs | HCB sign demonstrated in Figure 7 of article | Cerebellar and brainstem atrophy | MSA-C |
| 1. Gulati (2009) | The Hot Cross Bun Sign | India | Case report | N=2 | 45; 60 | 1M; 1F | Ataxia Parkinsonism | HCB sign demonstrated on transverse T2W MRI of pons | Atrophy of the putamen and brainstem and  Hyperintense signal in MCPs | MSA-C |
| 1. Pan et al (2015) | ‘Hot cross bun’ sign with leptomeningeal  metastases of breast cancer: a case report and  review of the literature | China | Case Report | N=1 | 58 | F | Drowsiness, confusion, slurred speech, involuntary movements | cruciform hyperintensity in the pons on T2W imaging | Linear enhancement in the cerebellar sulcus  Mass effect noted in the pons and midbrain | Leptomeningeal metastases |
| 1. Brooks et al (2018) | Hot-cross bun sign as a neuroradiological clue to multiple  system atrophy with predominant cerebellar ataxia | Brazil | Case Report | N=1 | 36 | F | Ataxia Dysautonomia | HCB sign seen on T2FLAIR | Cerebellar and pontine atrophy | MSA-C |
| 1. Chandran et al (2007) | Hot-cross bun sign | India | Case Report | N=1 | 60 | M | Ataxia Dysautonomia  Parkinsonism | Cruciform hyperintensity in the pons noted on T2W imaging | Cerebellar atrophy Dilatation of the 4^th^ ventricle | MSA-C |
| 1. Zhang et al (2013) | The "Hot Cross Bun" Sign in Leptomeningeal Carcinomatosis | China | Case report | N=1 | 38 | F | Vomiting, right VI nerve palsy, horizontal nystagmus | Hyperintense lesions in the central pons on T1W imaging  HCB sign noted on T2W imaging | Atrophy in the cerebellum and pons  No enhancement. | Leptomeningeal carcinomatosis Seizures |
| 1. Nagpal et al (2017) | “Hot-cross bun” and “inverse trident sign' in neurosarcoidosis: An important finding | India | Case report | N=1 | 48 | M | Pancerebellar syndrome  Pyramidal Signs | T2W imaging showed HCB sign in the pons with asymmetrical hyperintensities in the superior cerebellar peduncles | Axial T2W imaging at the upper pontine level showed the “inverse trident” sign | Neurosarcoidosis |
| 1. Ando et al (2021) | The hot cross bun sign in corticobasal degeneration | Japan | Case Report | N=1 | 61 | F | Ataxia. Dysautonomia  Cognitive impairment  Parkinsonism | Delayed HCB sign in the pons 5 years; noted only after onset | Atrophy of the brain stem, cerebellum, frontotemporal cortex, and corpus callosum  Dilated third and fourth ventricle White matter hyperintensity of the frontal lobes | CBD  (Autopsy case) |
| 1. Roh et al (2013) | Hot Cross Bun Sign Following Bilateral Pontine Infarction: A Case Report | Korea | Case Report | N=1 | 71 | F | Ataxia. | Imaging findings below occurred 1 year after the infarction. T2W imaging showed the HCB sign; pontine and cerebellar atrophy noted  Chronic infarcts in bilateral MCPs | Acute imaging findings of infarctions in bilateral pons and right cerebellum Severe occlusion of both vertebral arteries noted on MRA | Bilateral pontine infarction |
| 1. Koh et al (2008) | Cruciform Pontine MRI Hyperintensities (“Hot Cross Bun” Sign) in Non-Multiple System Atrophy Patients | Korea | Case report | N=1 | 61 | M | Gait disturbance | Cruciform signal hyperintensities within the pons and atrophy of pons | Delayed imaging 7 years following cerebellar haemorrhage | Cerebellar Haemorrhage |
| 1. Dalton et al (2020) | “Hot Cross Bun” Sign In Multiple System Atrophy | Ireland | Case Report | N=1 | 48 | F | Ataxia Dysautonomia Parkinsonism | HCB sign seen in pons on T2W and T2 FLAIR imaging | Nil else noted | MSA-C |
| 1. Moulignier et al (2015) | HIV-Associated JC Virus–Granule-Cell Neuronopathy (JCV–GCN) with the Hot-Cross-Bun Sign | France | Case Report | N=1 | 52 | M | Ataxia.  No dysautonomia or Parkinsonism. | HCB sign seen on T2W imaging | Cerebellar and pontine atrophy | JCV Granule cell neuronopathy  HIV infection |
| 1. Oji et al (2013) | Cerebellar ataxia and the “hot cross bun” sign in association with Human Immunodeficiency Virus infection | Japan | Case Report | N=1 | 32 | M | Cerebellar ataxia | Delayed imaging 1 year later showed cruciform hyperintensity in pons on T2W and T2FLAIR imaging | Left MCP hyperintensity  Cerebellar atrophy Fourth ventricle dilatation | HIV infection.  JC virus negative |
| 1. Henry et al (2015) | JC virus granule cell neuronopathy: A cause of infectious cerebellar degeneration | France | Retrospective review | N=5 | 31 - 43 | 1  M  4 F | Ataxia | HCB seen in 3 patients | Cerebellar and pontine atrophy | HIV infection.  Likely JC virus infection |
| 1. Yadav et al (2011) | "Hot cross bun" sign in HIV-related progressive multifocal leukoencephalopathy | India | N=2 | 29-F 32-M | 29  32 | F  M | Spinocerebellar syndrome with bulbar signs | T2, FLAIR, 3T MRI system showed the classical HCB sign | Reduced NAA peak and NAA/Cr ratio in cerebellum | HIV infection  JC virus infection |
| 1. Jain et al (2014) | 'Hot-cross bun' and 'inverse trident sign' in progressive multifocal leukoencephalopathy with HIV seropositivity | India | Case report | N=1 | 42 | M | Ataxia  Dysphagia | HCB sign noted in the middle and lower pons | 'Inverse trident sign' seen in the upper pons. Asymmetric T2W hyperintensities in the middle and inferior cerebellar peduncles and bilateral parieto-occipital regions Encephalomalacia | HIV  PML |
| 1. Jain et al (2013) | ‘Hot cross bun’ sign in a case of cerebrotendinous xanthomatosis: a rare neuroimaging observation | India | Case report | N=1 | 25 | M | Ataxia Quadriparesis Xanthoma  Cognitive decline | HCB sign seen in the pons | T1W hypointensities and T2W hyperintensities noted in the cerebellum bilaterally and the periventricular white matter | Cerebrotendinous xanthomatosis |
| 1. Bhudram (2017) | The ‘across the pons’ sign: A possible novel radiographic finding in natalizumabassociated progressive multifocal leukoencephalopathy | Canada | Case report | N=1 | 36 | F | Ataxic gait, dysdiadochokinesia | HCB sign seen in the pons | Right cerebellar white matter T2W hyperintensity with patchy enhancement | MS treated with Natalizumab  PML |
| 1. Das et al (2016) | ‘Hot cross bun’ sign | India | Case report | N=2 | 46; 64 | 2 M | Ataxia  Dysautonomia  Parkinsonism | HCB sign | Pontine, MCP and cerebellar atrophy | MSA-C |
| 1. Gan et al (2018) | The hot cross bun sign in a patient with encephalitis | China | Case report | N=1 | 3 | M | Skin rash, impaired vision, deafness, dysphagia, lower limb movement disorder, urinary incontinence  Clinical recovery following Immunmodulatory treatment | HCB sign noted at presentation. This resolved on follow up imaging 1 year later | No significant atrophy or enhancement | Viral encephalitis 1 month prior to presentation |
| 1. Ishikawa et al (2021) | Long-term MRI changes in a patient with Kelch-like protein 11-associated paraneoplastic neurological syndrome | Japan | Case report | N=1 | 42 | M | Cerebellar dysfunction  Sensorineural deafness | HCB sign | Brainstem and cerebellar atrophy  Low-signal intensity on SWI in the substantia nigra, red nucleus and dentate nuclei | Seminoma  Kelch-like protein 11 (KLHL11)-associated paraneoplastic neurological syndrome |
| 1. Liu et al (2021 | Neurological Autoimmunity Associated With Homer-3 Antibody | China | Case series | N=2 | 65; 50 | 1 M 1F | Ataxia  Dysautonomia | Delayed appearance of HCB sign | Delayed appearance of atrophy of the cerebellum and pons | Autoimmune cerebellar ataxia - Homer-3 antibodies |
| 1. Rissardo et al (2019) | Differential Diagnosis of Hot Cross Bun Sign | Brazil | Case report | N=1 | 59 | M | Ataxia Dysautonomia Parkinsonism | HCB sign seen on T2W and T2FLAIR | Pontine and cerebellar atrophy  Putaminal rim sign | MSA-C |
| 1. Padmanabhan et al (2013 | Hot cross bun sign in HIV‑related progressive multifocal leukoencephalopathy | India | Case Report | N=1 | 37 | F | Ataxia | HCB sign in pons | Asymmetric left middle and inferior cerebellar peduncles T2W hyperintensities extending into left cerebellar hemisphere with atrophy of the same structures | HIV  PML |
| 1. Ilyas et al (2016) | Multiple System Atrophy-Cerebellar Type (MSA- C): A Case Report | India | Case Report | N=1 | 58 | M | Ataxia  Pyramidal signs | HCB sign in pons | Atrophy of pons, middle cerebellar peduncles, the cerebellum, inferior cerebellar peduncle and inferior olivary nucleus  Diffuse cortical atrophy | MSA-C |
| 1. Takao et al (2007) | ‘Hot-cross Bun Sign’ of Multiple System Atrophy | Japan | Case Report | N=1 | 43 | M | Ataxia. Dysautonomia. Parkinsonism. | HCB at the level of pons on T2WI | Nile else noted | MSA-C |
| 1. Recio Bermejo M, et al (2012) | ‘‘Hot-cross bun sign’’ in multiple system atrophy: A presentation of 3 cases | Brazil | Case report | 62- F  74-F  85-M | 62-  74  85 | 1M 2 F | Patient 1- Ataxia. Dysautonomia.  Parkinsonism  Patient 2 - Ataxia. Parkinsonism.  Patient 3 - Ataxia. Dysautonomia. Parkinsonism. | HCB sign in pons | Nil else reported | MSA-C |
| 1. Pérez Errazquin et al (2010) | Sign of the cross and MSA-C | Spain | Case Report | N=1 | 50 | M | Ataxia. Dysautonomia. Parkinsonism. | Hot cross bun sign noted | Cerebellar atrophy  Putaminal rim sign  Hypointense putamen | MSA-C |
| 1. Vijayan et al (2008) | MR imaging in multiple system atrophy: Its role in “splitting” parkinsonism | India | Case report | N=1 | 59 | F | Ataxia. Dysautonomia. Parkinsonism. | T2/ PD/FLAIR sequences demonstrated the HCB sign | Hyperintensity of bilateral MCP  Putaminal, pontine and cerebellar atrophy | MSA-C |
| 1. Belenciuc et al | Cerebellar ataxia and the “Hot cross bun” sign in association with human immunodeficiency virus infection - A case report and review of literature | Moldova | Case report | N=1 | 33 | M | Cerebellar signs  Pyramidal signs | HCB sign in middle and lower pons | Asymmetric T2-weighted hyperintensities in middle cerebellar peduncles and pons. No enhancement noted | HIV  CD4 210 |
| 1. Way et al (2019) | The ‘Hot Cross Bun’ Sign Is Not Always Multiple  System Atrophy: Aetiologies of 11 Cases | USA | Retrospective review | N=11 | 40 – 79 | 5 M 6F | Cognitive decline and hallucinations. Parkinsonism.  Ataxia  Dysautonomia | HCB sign noted | Nil else noted | DLB (n=1)  Possible/ ProbableMSA (n=6)  Hereditary Ataxia (n=3)  Unkown (n=1) |
| 1. Zhu et al (2020) | Various Diseases and Clinical Heterogeneity Are Associated With “Hot Cross Bun” | China | Mixed Methods Retrospective review  Systematic review | N=79 | 13 -71 | 30 M 49 F | Ataxia  Parkinsonism  Dysautonomia  Pyramidal signs  Cognitive decline  Epilepsy  Loss of weight | Hot cross bun signs seen on T1, T2 and T2W FLAIR imaging | MCP hyperintensity was more frequent in the neoplastic and degenerative groups.  Pontine, MCP and cerebellar atrophy was noted mostly in the hereditary and neurodegnerative groups  HCB was noted to progress in neurodegnerative disease, but improve in inflammatory diseases | Not previously described  Inflammation – NMOSD, ADEM, MS  Neoplastic – Lung Cancer  Toxic – Phenytoin toxicity  Brainstem encephalitis |
| 1. Lin et al (2016) | A Case of Multiple System Atrophy with Pre-existing Alzheimer’s Disease and Predating The Hot Cross Bun Sign | Taiwan | Case Report | N=1 | 69 | M | Ataxia  Mild Parkinsonism  Dysautonomia  Cognitive decline | HCB sign noted on T2W imaging | HCB sign predated the diagnosis of MSA-C  MCP hyperintensity  Pontine, MCP cerebellum atrophy | Alzheimer Dx  MSA-C |
| 1. Srivastava et al (2005) | “Hot cross bun” sign in two patients with multiple system atrophy-cerebellar | India | Case report | N=1 | 60  56 | F  M | Ataxia  Parkinsonism  Dysautonomia  Pyramidal signs | T2W hyperintensity in the pons – HCB sign | Pontocerebellar atrophy  MCP atrophy  Medullary atrophy | MSA-C |
| 1. Shiraishi et al (2021) | A Unilateral Bright Middle Cerebellar Peduncle Sign | Japan | Case Report | N=1 | 62 | M | Ataxia  Dysautonomia | HCB sign | Left MCP hyperintense on T2W and T2FLAIR imaging | MSA-C |
| 1. Constantinides et al (2021) | Hot cross bun sign and prominent cerebellar peduncle involvement in a patient with oculodentodigital dysplasia | Italy | Case Report | N=1 | 42 | M | Digital Dysplasia  Small pupil size  Nasal Dysplasia  Microdontia  Dysplastic ears prognathism  Hyperreflexia  L SNHL  R Conductive HL | HCB sign | T2W white matter changes in the parietal and occipital lobes  Corticospinal tracts hyperintense on T2FLAIR  Superior and middle cerebellar peduncles markedly hyperintense in T2  Hypointense basal ganglia and thalami | Oculodentodigital dysplasia |
| 1. Rohani et al | Hot cross bun sign in a case with multisystem atrophy | Iran | Case report | N=1 | 65 | M | Ataxia  Dysautonomia | Hot cross bun sign in pons | Cerebellar and pontine atrophy | MSA-C |
| 1. Bhat et al (2020) | Neuroimaging Findings in Rabies Encephalitis | India | Case report | N=1 | 28 | F | Encephalitis  Paralysis | HCB sign in pons | Dentate nuclear hyperintensity. Basal ganglia, midbrain and spinal cord gray matter T2 hyperintensity Contrast enhancement | Rabies encephalitis |
| 1. Majed et al (2022) | “Hot Cross Bun” Sign in a Patient With Kelch-like Protein 11 Rhombencephalitis | USA | Case report | N=1 | 36 | M | Ataxia  Parkinsonism  Hearing Loss  Diplopia | Cruciform T2 hyperintensity in pons | T2 hyperintensity of MCPs Atrophy of the brainstem and cerebellum | Seminoma |
| 1. Zhu et al (2021) | “Hot cross bun” is a potential imaging marker for the severity of cerebellar ataxia in MSA-C | China | Prospective Cohort study | N=81 | MSA – C = 53  MSA – P= 58 | 30 M  51 F | Ataxia  Parkinsonism  Dysautonomia | HCB Grade 3 | Atrophy of pons  Atrophy of MCP | MSA-C n = 50  MSA-P n = 31 |
| 1. Deguchi et al (2015) | Significance of the hot-cross bun sign on T2*-weighted MRI for the diagnosis of multiple system atrophy | Japan | Retrospective review | N=54  MSA-C = 33  MSA-P= 21 | 48 - 83 | 29 M  25 F | Ataxia  Parkinsonism  Dysautonomia | MSA-C – 32/33 (96%) HCBS  MSA-P – 13/21 (38%) HCBS | HCB sign was more easily seen in MSA-C than MSA-P  Putaminal atrophy  Putaminal hyperintense rim | MSA |
| 1. Horimoto (2002) | Longitudinal MRI study of multiple system  Atrophy – when do the findings appear,  And what is the course | Japan | Retrospective review | 41- 74 | 26 Male 16 Female | 42 | Parkinsonism  Dysautonomia  Cerebellar signs | HCB sign | Putaminal slit sign | MSA A, P, C |
| 1. Abe (2006) | The “Cross” Signs in Patients With Multiple System Atrophy: A Quantitative Study | Japan | Retrospective review | 40 to 72 | 39 men 29 women | 68 | MSA C, P  Ataxia, Parkinsonism | HCB sign | Atrophy of the pontine base and cerebellar vermis | MSA C, P |
| 1. Schott (2003) | Delineating the Sites and Progression of In Vivo Atrophy in Multiple System Atrophy Using Fluid-Registered MRI | UK | Case report | 60 | Male | 1 | Ataxia, autonomic symptoms, obstructive sleep apnoea, memory decline, Parkinsonism, pyramidal signs | HCB sign | MCP and cerebellar atrophy | MSA |
| 1. Lo Coco (2003) | How specific are the pontine MRI hyperintensities (the cross sign)? | Italy | Case Report | 41 | Female | 1 | Diplopia | HCB sign | No infratentorial atrophy noted | MG |
| 1. De Albuquerque (2007) | Multiple System Atrophy – Clinical-Radiological Correlation  Report of two cases | Brazil | Case Report | 57 (Male)  60 (Female) | 1 male 1 female | 2 | Case 1 - Autonomic dysfunction, cerebellar ataxia, pyramidal signs  Case 2 - asymmetrical Parkinsonism, autonomic signs, pyramidal signs, insomnia | HCB sign | Brainstem, cerebellar atrophy | MSA |
| 1. Savoiardo (1990) | Olivopontocerebellar atrophy – MR diagnosis and relationship to Multiple Systems Atrophy | Italy | Retrospective review | 33 - 64 years | 13 male 10 female | 23 | OPCA, extrapyramidal disorder, autonomic signs | HCB sign | Brainstem and cerebellar atrophy | OPCA with MSA, OPCA without MSA |
| 1. Pradhan (2017) | Relevance of non-specific MRI features in multiple system atrophy | India | Retrospective review | Mean age: 59.53 ± 9.74 years | 38 male 15 female | 53 | Parkinsonism, autonomic failure, cerebellar signs | Hot cross bun sign | Atrophy of the midbrain, pons, cerebellum, MCP, corpus callosum, putamen  Hyperintense putamen rim  MCP hyperintensity  Putaminal hypointensity | MSA |
| 1. Alsemari et al (2016) | Large-scale mitochondrial DNA deletion underlying familial multiple system atrophy of the cerebellar subtype | Saudi Arabia | Case Series | N=1 | 33 | F | Ataxia  Dysautonomia | Faint hot cross bun sign in noted in the pons | Cerebellar atrophy with mild pontine and medulla oblongata atrophy | MSA-C |
| 1. Baronica et al (2011) | Differential Diagnostic Relevance of High Resolution Magnetic Resonance in Patients with Possible Multiple System Atrophy (MSA) – A Case Report | Croatia | Case Report | N=1 | 60 | M | Ataxia  Parkinsonism  Dysautonomia | HCB sign | Brain stem, cerebellum and basal ganglia atrophy  Putaminal slit sign  MCPs hyperintense | MSA-C |
| 1. De Mello et al (2010) | Multiple-System Atrophy with Cerebellar Predominance Presenting as Respiratory Insufficiency and Vocal Cords Paralysis | Portugal | Case Report | N=1 | 79 | F | Dyspnoea, asthenia, stridor, and respiratory failure. Dysautonomia  Ataxia | HCBS on T2W | Atrophy of MCPs, pons and cerebellum | MSA-C |
| 1. Doan et al (2021) | Video Representation of Dopamine-Responsive Multiple System Atrophy Cerebellar Type | USA | Case report | N=1 | 61 | M | Ataxia  Parkinsonism  Asthenia  Pyramidal signs  Sensory neuropathy | HCBS | Atrophy of the pons, MCPs and cerebellum | MSA-C |
| 1. Hohler et al (2012) | Probable hereditary multiple system atrophy–autonomic (MSA–A) in a family in the United States | USA | Case report | N=1 | 84 | F | Dysautonomia | HCBS | Small left pontine stroke | MSA-A |
| 1. Massey et al (2012) | Conventional Magnetic Resonance Imaging in Confirmed Progressive Supranuclear Palsy and Multiple System Atrophy | UK | Retrospective review | N=13 | 52.9 | 6= F  7 = M | 10 cases - predominantly Parkinsonian  2 cases - predominantly Cerebellar  1 case- purely autonomic | MCP sign  HCB sign | Atrophy of the putamen, pons, MCPs and cerebellum | MSA |
| 1. Muñoz-Lopetegi et al (2021) | Magnetic resonance imaging abnormalities as a marker of multiple system atrophy in isolated rapid eye movement sleep behaviour disorder | Spain | Prospective cohort | N=2 | 70 ,60 | 1M 1F | Ataxia  Parkinsonism  Parkinsonism  RBD | HCB sign. | Putaminal rim sign  Atrophy of the pons, MCPs and cerebellum | MSA-C |
| 1. Goldman et al (2014) | Multiple System Atrophy and Amyotrophic Lateral Sclerosis in a Family with Hexanucleotide Repeat Expansions in C9orf72 | USA | Case Report | N=1 | 65 | F | Ataxia  Dysautonomia  Parkinsonism | HCB sign | Pontine and cerebellar atrophy | MSA-C |
| 1. Zhao et al (2020) | Clinical features, MRI, and 18F-FDG-PET in differential diagnosis of Parkinson disease from multiple system atrophy | China | Prospective Cohort | N=28 | 58.82 | 12 – M  16 - F | Ataxia  Dysautonomia  Parkinsonism | HCB sign | Cerebellar atrophy  MCP hyperintense signal | MSA-C |
