# Supplementary material for: The “Hot Cross Bun Sign” in Spinocerebellar Ataxia Types 2 and 7–Case Reports and Review of Literature
Source: Mov Disord Clin Pract. 2022 Oct 13;9(8):1105–13. doi: 10.1002/mdc3.13550 (PMC9631856; doi:10.1002/mdc3.13550)
Supplement: Supplementary file 2 — Table S2 Scoping review of the Hot Cross Bun Sign in the Spinocerebellar Ataxias [file MDC3-9-1105-s001.docx]

| **Supplementary Table 2. Scoping Review of the Hot Cross Bun Sign in the Spinocerebellar Ataxias** | | | | | | | |
| --- | --- | --- | --- | --- | --- | --- | --- |
| **Author and date** | **Title of Study** | **Aim of Study** | **Study Setting: Country/ Region** | **Study Population** | **Data Collection Methods** | **SCA Type** | **Results of Study** |
| 1. Higashi et al   2017 | Cerebellar ataxia subgroups can be differentiated by pontine magnetic resonance imaging | To clarify usefulness of pontine T2W MRI to pinpoint SCA subgroups - spinocerebellar ataxia with brainstem involvement (SCA-BSI), pure cerebellar SCA, and cerebellar dominant multiple system atrophy (MSA-c) | Tokyo Japan | 208 patients | Single institution cross sectional-Prospective study | SCA 2 | HCBS was a highly specific marker for MSA-C, with a sensitivity of 67.6%. HCBS was also observed in 60% of SCA2 cases and was absent in all cases of MJD, SCA1, and DRPLA |
| 1. Ozaki et al 2017 | Ataxia and Cerebellar Disorders – Clinico-radiological characteristics of SCA34 patients with the hot cross bun sign caused by the P.TRP246GLY mutation in ELOVL4 | To present detailed clinic-radiological characteristics of SCA34 patients with the mutation, p.Trp246Gly, in ELOVL4 | Japan | n=9 | Case Series | SCA 3 | MRI revealed  hot cross bun sign or pontine midline linear hyperintensity in  addition to pontine and cerebellar atrophy |
| 1. Higashi et al 2018 | A diagnostic decision tree for adult cerebellar ataxia based on pontine magnetic resonance imaging | Identifying the specificity and sensitivity of key MRI findings, such as HCBS and PMH, together with a reliable diagnostic flow chart. Investigated a novel marker, ‘BT-ratio’ which represents; the distance ratio of the pontine base to the tegmentum. | Tokyo, Japan | n=22 | retrospective analysis was performed on consecutive patients with SCA | SCA-1, SCA-2 | The presence of HCBS indicated either MSA-C or SCA2. |
| 1. Aralasmak et al 2010 | Imaging in Neurodegenerative Disorders | To discusses imaging findings of the cortical and subcortical neurodegenerative  diseases | Turkey | N/A | Review article | SCA 1, SCA 2 | HCB sign is detected in MSA-C, SCA 1 and SCA2 |
| 1. Heidelberg et al 2018 | Main inherited neurodegenerative cerebellar ataxias, how to recognize them using magnetic resonance imaging? | To discuss a pattern recognition approach to help neuroradiologists differentiate the most frequent profiles of ataxia | France | N/A | Review article | SCA6,  SCA 7 | SCA 7 can demonstrate the HCB sign  SCA 6 can demonstrate a midline linear pontine T2W hyperintensity in late disease |
| 1. Sheng Lin et al 2007 | The SCA17 phenotype can include features of MSA-C, PSP and cognitive impairment | Report of a new phenotype of SCA17 | Taiwan | n=1 | Case Report | SCA 17 | A ‘‘hot cross bun’’ sign in pons was noted |
| 1. Alvarenga et al 2021 | Frequency and features of spinocerebellar ataxias in Brazilian  patients from Rio de Janeiro | To describe  the frequency and features of SCAs in a cohort of patients from Rio de Janeiro | Rio De Janeiro | n=147 | Retrospective study | SCA 3  SCA 7, SCA 2, SCA 1, SCA 6 and  SCA 8 and SCA 10 | SCA 3 was the most frequent type. Less frequent types were SCA 7, SCA 2, SCA 1, SCA 6 and  SCA 8 and SCA 10  HCB sign seen in 15% of cases (SCA subtype not stated). |
| 1. Cocozza et al 2021 | Conventional MRI findings in hereditary degenerative ataxias: a pictorial review | Review of the main clinical and conventional imaging findings of the most common hereditary degenerative ataxias | Italy | n/a | Review | SCA 7,  SCA 8 | The HCB sign was noted in one patient each with SCA 7 and SCA 8 (Lee; see below) |
| 1. Gooneratne et al 2013 | Hot cross bun sign in a patient with cerebellar ataxia | To demonstrate the HCB sign in a case of genetically confirmed SCA2 | Sri Lanka | n=1 | Case Report | SCA2, | Hot Cross Bun Sign in SCA 2 |
| 1. Haeri et al 2021 | Congenital Ichthyosis in a Case of  Spinocerebellar Ataxia Type 34: A Novel  Presentation for a Known Mutation | Describe a case and literature review of SCA 34 | Iran | n=1 | Case Report | SCA 34 | MRI revealed the hot cross bun sign in  addition to pontocerebellar atrophy |
| 1. Indelicato et al 2018 | Autonomic function testing in spinocerebellar ataxia type 2 | To assess whether autonomic failure belongs to the clinical spectrum of spinocerebellar ataxia type 2 (SCA2 | Austria | n=8 | Case Series | SCA 2 | Two patients showed a hot-cross-bun sign |
| 1. Khadilkar et al 2012 | Trinucleotide repeat spinocerebellar ataxias:  experience of a tertiary care centre in Western  India with review of Indian literature | To present the frequency of SCA mutations in a tertiary care  centre in Western India | India | n=30 | Case Series | SCA 6 | Most common was SCA  2 (10 patients). SCA1, SCA 3 and SCA 6 also detected.  HCB sign only detected in the SCA 6 case. |
| 1. Kim et al 2019 | Differential value of brain magnetic  resonance imaging in multiple  system atrophy cerebellar  phenotype and spinocerebellar  ataxias | To assess the prevalence of HCB signs and MCP changes in brain MRIs based on the duration of MSA-C and SCAs | Korea | n=186 with MSA n=117 with SCA | Retrospective review | SCA 2, 3, 7, 8 | HCB Sign is not found early in the course of SCA (< 3 years since disease onset)  SCA 1, 6 and 17 did not demonstrate HCB sign |
| 1. Lee et al 2009 | The hot cross bun sign in the patients with spinocerebellar ataxia | Investigate the  frequency of HCBS in the patients with spinocerebellar ataxia (SCA) and healthy  controls. | Taiwan | n=138 SCA | Retrospective review | SCA 2,3,7,8 | HCB sign was found mostly in SCA 2 cases. Only one case each of SCA 3, 7 and 8 demonstrated a HCB sign  SCA 1, 6 and 17 did not show HCB sign |
| 1. Li et al 2018 | A case of a novel CACNA1G mutation from a  Chinese family with SCA42 | Presented a novel genetic mutation in 3 patients with SCA42 | China | n=3 | Case Report | SCA 42 | cerebellar atrophy,  and the hot cross bun sign of brainstem was found in 2 patients |
| 1. Meira et al 2019 | Neuroradiological Findings in the Spinocerebellar Ataxias | Review the particular neuroradiological abnormalities in the main SCAs. | USA | N/A | Literature review | SCA 1, 2, 3, 6, 7, 8, 34 | HCB sign detected in SCA 1, 2, 3, 6, 7, 8, 34  Multiple other SCA subtypes described, but did not show HCB sign |
| 1. Moreno-Escobar et al 2022 | Hot Cross Bun Sign in Progressive Ataxia  with ELOVL4 Mutation—Case Report | To discuss the function of the protein ELOVL4, mutation of which causes SCA34 | USA | N=1 | Case Report | SCA34 | HCB sign seen on MRI |
| 1. Namekawa et al 2015 | “Hot Cross Bun” Sign Associated with SCA1 | First reported case of HCB sign in SCA1 | Japan | n=2 | Case Report | SCA 1 | HCB sign detected at lower pontine level |
| 1. Ozaki et al 2015 | A Novel Mutation in *ELOVL4* Leading to Spinocerebellar  Ataxia (SCA) With the Hot Cross Bun Sign  but Lacking Erythrokeratodermia  A Broadened Spectrum of SCA34 | To identify the causative gene of SCA in 2 Japanese families with distinct  neurological symptoms and radiological presentations. | Japan | n=11 | Clinical genetic study | SCA 34 | HCB sign seen in 6 cases; midline pontine linear hyperintensity seen in 2 cases |
| 1. Ozaki et al 2019 | Prevalence and clinic-radiological features of spinocerebellar ataxia type 34  in a Japanese ataxia cohort | Detection of SCA34 by mutation screening of 153 patients with degenerative ataxias | Japan | n=153 | Genetic Study | SCA 34 | 2 cases of SCA 34 detected; MRI of both patients showed HCB sign |
| 1. Pedroso et al 2012 | “Hot cross bun” sign resembling multiple  system atrophy in a patient with  Machado-Joseph disease | To describe “Hot cross bun sign” in MJD. | Brazil | n=1 | Case Report | SCA 3 | Genetic testing was positive for Machado-Joseph disease  HCB sign was demonstrated; it is an uncommon finding in SCA3 |
| 1. Wang et al 2021 | Diagnostic efficacy of the magnetic resonance  T1W/T2W ratio for the middle cerebellar  peduncle in multiple system atrophy and  spinocerebellar ataxia: A preliminary study | investigate the diagnostic value of the MCP sT1w/T2w ratio for differentiating  between MSA-C and spinocerebellar ataxia (SCA). | Japan | N=32 MSA-C, n= 8 SCA type 3 (SCA3), n= 16 SCA type 6 (SCA6) patients, n= 17 controls | Retrospective study | SCA 3 | HCB sign was rare in SCA3; disease duration for SCA ranged from 0.8 to 5.7 years.  HCB was more frequently seen in MSA  HCB sign was not observed in SCA6  The MCP sT1W/T2W ratio was useful to differentiate MSA from SCA; it is lower in MSA. |
| 1. Saigoh et al 2015 | The first Japanese familial case of spinocerebellar ataxia 23 with a  novel mutation in the PDYN gene | First reported case of familial SCA 23 in Japan | Japan | n=1 | Case report | SCA 23 | HCB sign seen on MRI |
| 1. Soto et al 2020 | Pontine hot cross bun sign in spinocerebellar  ataxia type 2 | Description of HCB sign in SCA2 | Canada | n=1 | Case Report | SCA 2 | severe pontocerebellar atrophy  with hot cross bun sign |
| 1. Sugiyama et al 2019 | Diagnostic Challenges Posed by Preceding Peripheral  Neuropathy in Very Late-onset Spinocerebellar  Ataxia Type 3 | Report of a case of very late onset SCA3; cerebellar signs were masked by peripheral neuropathy | Japan | n=1 | Case Report | SCA 3 | midline linear  high-intensity area was noted in the pons, heralding the development of the HCB sign |
| 1. Sugiyama et al 2020 | Vertical pons hyperintensity and hot cross  bun sign in cerebellar-type multiple system  atrophy and spinocerebellar ataxia type 3 | study compared the frequency and timing of HCB appearance in the disease course between patients with MSA (either parkinsonian or cerebellar) and with SCA3 | Japan | n=80 | Retrospective study | SCA 3 | HCB is a highly sensitive finding for MSA-C, even in the early stages of the disease.  The HCB sign develops much later in SCA3 compared to MSA |
| 1. Wang et al 2016 | Hot cross bun sign in a late-onset SCA1 patient | Case of late onset SCA1 | Japan | n=1 | Case Report | SCA 1 | Prominent HCBS in the pons  and severe atrophy in both the cerebellum and the  brainstem |
| 1. Way et al 2019 | The ‘Hot Cross Bun’ Sign Is Not Always Multiple  System Atrophy: Aetiologies of 11 Cases | clarify the specificity of the ‘hot cross bun’ sign (HCBS) for multiple system atrophy (MSA) in adult cerebellar  ataxia or parkinsonism. | Korea | n=11 | Retrospective review | Hereditary Ataxia, aetiology unknown | 3 cases of hereditary ataxia demonstrated the HCB sign.  Genetic testing was negative (SCA 1, 2, 3 and 6 in all three cases plus SCA 7 and/or 8 and/or 10 and/or 17) |
| 1. Yamasaki et al 2017 | Early strong intrathecal inflammation in  cerebellar type multiple system atrophy by  cerebrospinal fluid cytokine/chemokine  profiles: a case control study | aimed to characterize CSF cytokine/  chemokine/growth factor profiles in MSA-C and compare  them with hereditary SCA to determine correlations  between CSF cytokine/chemokine/growth factor  profiles and disease stages, clinical severity, and brain atrophy  in MSA-C | Japan | n=47 | Prospective study | SCA 6, 8, 31  Hereditary Ataxia, aetiology unknown | HCB sign identified on MRI. However, specific details reflecting SCA subtype not disclosed |
| 1. Zhu et al 2020 | Various Diseases and Clinical  Heterogeneity Are Associated With  “Hot Cross Bun” | To characterize the clinical phenotypes associated with the “hot cross  bun” sign (HCBs) on MRI and identify correlations between neuroimaging and  clinical characteristics | China | n=79 | Retrospective review and systematic review | SCA; subtypes not reported | HCB sign noted in SCA. However, subtypes not reported |
| 1. Koh et al 2008 | Cruciform Pontine MRI Hyperintensities (“Hot Cross Bun” Sign) in Non-Multiple System Atrophy Patients | Describe HCB in non MSA patients | Korea | n=2 | Case Report | SCA 2 | One case of SCA2 noted; this patient’s MRI showed HCB sign |
